# Genomic Landscape of High‐Altitude Adaptation in East African Mountain Honey Bees ( *Apis mellifera* )

**DOI:** 10.1002/ece3.71846

**Published:** 2025-08-20

**Authors:** Marco Mazzoni, Florian Loidolt, Sonja Kersten, Deborah Ruth Amulen, Patrick Vudriko, Philipp Meyer, Victor Sebastian Scharnhorst, Ricarda Scheiner, Martin Hasselmann

**Affiliations:** ^1^ Department of Livestock Population Genomics, Institute of Animal Science University of Hohenheim Stuttgart Germany; ^2^ Behavioural Physiology and Sociobiology, Biocenter Universität Würzburg Würzburg Germany; ^3^ The College of Veterinary Medicine Animal Resources and Bio‐security (COVAB) Makerere University Kampala Uganda; ^4^ Institute for Integrative Nature Conservation Research University of Natural Resources and Life Sciences Vienna Vienna Austria; ^5^ KomBioTa‐Center for Biodiversity and Integrative Taxonomy University of Hohenheim and State Museum of Natural History Stuttgart Germany

## Abstract

Understanding the evolutionary processes leading to differentiation within species is a central goal in population biology. A key process is local adaptation, for which organisms evolve traits enhancing the survival and reproduction in specific environments. Honey bees ( *Apis mellifera* ) in East Africa are well adapted to highland environments, showing different phenotypes, including behavior, compared to lowland bees. Despite these differences, highland and lowland honey bees show very low genetic differentiation, with the exception of two segments on chromosome 7 (r7) and chromosome 9 (r9), which were previously identified as chromosomal inversions. These inversions are rare in lowland populations, suggesting a key role in adaptation to high‐elevation habitats. In this study, we obtained 24 whole genomes from honey bees of Western Uganda and compared these with existing data from Kenya. We show that the chromosomal inversions play a pivotal role in local adaptation in both regions but with substantial differentiation. Genome‐wide analysis of polymorphism revealed additional genomic regions potentially involved in high‐altitude adaptation. The acquisition of transcriptome data from highland and lowland honey bees in Uganda has enabled the first insights into the differential expression of genes between these bees. Our findings elucidate the involvement of genes in behavioral and oxygen consumption processes. This paves the way to clarify the interplay of r7 and r9 with gene expression and to unravel the regulatory network underlying *A. mellifera* adaptation to high‐elevation habitats. Our study will contribute to a better understanding of the evolutionary processes in honey bee populations driven by environmental conditions.

## Graphical abstract

Highland and lowland honey bees in East Africa exhibit distinct phenotypes despite low overall genetic differentiation. To investigate this further, we sampled honey bees from a mountain system in Uganda (red circle) and compared them with samples from Kenya. Our analyses confirmed that two chromosomal inversions (r7 and r9) were strongly associated with high‐altitude adaptation. Additionally, genome and transcriptome data revealed genes involved in behaviour and oxygen consumption, highlighting key genetic and regulatory mechanisms underlying local adaptation in *Apis mellifera*.

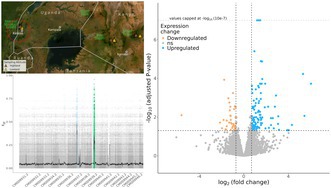

## 1 Introduction

Understanding the molecular mechanism that leads to the adaptation of organisms to different environments is a longstanding goal in evolutionary genetics (Goncearenco et al. 2013; Bomblies and Peichel 2022; Martínez Sosa and Pilot 2023; Byers et al. 2016). In light of ongoing climate change and decline of biodiversity (Franks and Hoffmann 2012; Mahecha et al. 2022), this knowledge is further valuable for the conservation of wild populations (Waldvogel et al. 2020; Webster et al. 2023). Adapting to different environments present numerous challenges for the organism (Matur et al. 2023). High mountain environments impose various stressors that can interfere with insect physiology, such as temperature, air density, and oxygen partial pressure (Dillon 2006). For example, it has been demonstrated that these stresses have the capacity to reduce metabolic rates and increase hatching times in *Manduca sexta* while also increasing the developmental time and reducing body size in *Tenebrio molitor* and *Drosophila melanogaster* (Greenberg and Ar 1996; Woods and Hill 2004; Dillon 2006). Although low temperatures relative to low altitudes may pose risks, evidence suggests that insects exposed to low oxygen levels prefer colder rather than warmer environments (Frazier et al. 2001).

Interestingly, studies in human populations residing in distinct high‐altitude regions have revealed a disparate pattern of selected genes, indicating that the process of adaptation can occur independently in each population (Scheinfeldt and Tishkoff 2010; Bigham et al. 2010; Bigham 2016).

Major implications of structural variants (SVs) have been identified in various adaptation processes. SVs can have an effect outside the boundaries of the SV, and this effect can influence gene expression, metabolic pathways, and finally speciation. Studies in *Drosophila* showed that the recombination in inversion polymorphisms can be extended even outside the inverted region (Stevison et al. 2011). Normally, linkage disequilibrium between two SNPs decays proportionally to the distance between the two; however, SVs, in particular inversions, disrupt this pattern, leading to extensive LD (Fuller et al. 2017). Inversions are a portion of DNA that is reversed end‐to‐end; they do not cause a loss of genetic material; however, they disrupt homologous pairing during meiosis. Crossing‐over events in heterozygous inversions can lead to the formation of abnormal chromatids. These are generally removed during gametogenesis, thus leading to the independent accumulation of genetic differences due to a lack of recombination. However, in some cases, double crossing‐over or gene conversion events could homogenize, at least in part, the genetic differences. This effect is observed only in the case of SNPs not strongly associated with divergent selection between populations (Feder and Nosil 2009; Korunes and Noor 2018). Finally, SVs can alter gene expression mainly through the disruption of genes at their breakpoints (Puig et al. 2004). There is substantial evidence that both small SVs, such as insertion and deletion, and large SVs, inversion and Copy Number Variants (CNVs), play a role in evolutionary and speciation processes (Schaeffer et al. 2003; Lin et al. 2017; Stalder et al. 2022). Indels in coding regions could cause a frameshift leading to changes in protein sequence and possibly lossoffunction of the protein (Lin et al. 2017). However, if the gene is not essential for the organism's fitness, it will just contribute to the phenotypic variability of the population. Inversion polymorphisms, particularly large ones, can capture different adaptive alleles and limit recombination between orientations, which can facilitate the fixation of these alleles in one or the other orientation. In some instances, such inversions may function as supergenes or superalleles and have been implicated in shaping the genetic basis of certain complex traits (Berdan et al. 2022; Gadau and Fewell 2022). Studies on inversion polymorphisms, which rely mainly on *Drosophila* sp., observed that inversion polymorphisms are associated with heat resistance, cold tolerance, and body size (Weeks et al. 2002; Bochdanovits and Jong 2003; Anderson et al. 2005). However, these studies also highlight that not every inversion should be considered as a superallele (Schaeffer et al. 2003).

In light of the continuous advancement and refinement of genome sequencing techniques, a growing body of evidence has emerged demonstrating the role of SVs in a variety of adaptive processes across a multitude of species. For example, in three‐spined sticklebacks ( *Gasterosteus aculeatus* ), inversion polymorphisms have facilitated colonization of both freshwater and marine ecosystems (Jones et al. 2012; Reid et al. 2021). In the yellow monkeyflower ( *Mimulus guttatus* ) an inversion polymorphism affected adaptive flowering time (Lowry and Willis 2010). Additionally, in deer mice ( *Peromyscus maniculatus* ), 13 inversions have been identified that contribute to differentiation between forest and prairie ecotypes, influencing traits related to local adaptation (Harringmeyer and Hoekstra 2022).

*Apis mellifera* , the western honey bee, is well adapted to a wide range of habitats and environments. It originated in Asia, where it diverged from its sister taxa, *Apis cerana* . From there, it colonized the Middle East, Africa, and Europe through various expansion events (Dogantzis et al. 2021). Each newly colonized environment poses threats to colony survival, making worker traits crucial for adaptation. Therefore, these traits often undergo positive selection in new areas (Harpur et al. 2014; Dogantzis et al. 2021). Honey bees maintain high genetic diversity, with more than 30 subspecies described, currently grouped into seven distinct lineages (Dogantzis et al. 2021). Among these, predominantly African honey bees in the A‐lineage represent the highest genetic diversity. In East Africa, two subspecies are identified around mountain peaks, *Apis mellifera scutellata* (*A. m. scutellata*) and *Apis mellifera monticola* (*A. m. monticola*; Ruttner 1998). *A. m. scutellata* inhabits the lowland savannah, while *A. m. monticola* inhabits the highland mountain forest environment (Smith 1961). Both environments pose severe threats to honey bees: the savannah is hot with scarce food sources, while the mountain forest is rainy, cold, and high‐altitude, causing potential hypoxia due to lower barometric pressure. The two subspecies exhibit differences in behavior and phenotype; *A. m. scutellata* is more aggressive, smaller, and brighter in color, whereas *A. m. monticola* is less aggressive, larger, and darker (Smith 1961; Ruttner 1998; Gruber et al. 2013).

Honey bee populations inhabiting East African regions are well adapted to both highland montane areas and lowland savannahs, with two inversion polymorphisms on chromosome 7 (named CM009937.2 in the latest *Apis mellifera* reference genome, Accession: GCA_003254395) and 9 (named CM009939.2 in the latest *Apis mellifera* reference genome, Accession: GCA_003254395), named r7 and r9 respectively, which are found at high frequency in the highland population (Wallberg et al. 2017; Christmas et al. 2018). The r7 (~591 Kbp) region harbors 27 genes and two lncRNAs while r9 (~1736 Kbp) contains nine genes, four microRNAs, one tRNA, and six lncRNAs. Both regions harbor potential candidate genes for the adaptation to high elevation habitats as previously described (Wallberg et al. 2017): r7 includes four out of five octopamine receptors (LOC413698, LOC412994 and LOC412896), which regulate complex behaviors such as division of labor, foraging, memory formation, and thermogenesis (Behrends and Scheiner 2012; Scheiner et al. 2014; Reim and Scheiner 2014; Kaya‐Zeeb et al. 2022). On the other hand, r9 includes a peripheral plasma membrane protein CASK (LOC411347), which is reported to interact with CaMKII and together promotes memory formation (Wallberg et al. 2017; Scholl et al. 2015) So far, these two inversions have been identified only in populations from three East African mountain systems (Mau region, Mt. Kenya, and Mt. Elgon). However, their presence in other East African mountains has yet to be confirmed. Everitt et al. (2023) examined high‐altitude populations in South America and found no evidence of these inversions there, suggesting that their role in altitude adaptation may be geographically restricted.

However, it is conceivable that not only genes within the two inverted regions are not the sole determinants of adaptation to high‐elevation habitats in East African regions, but that genome‐wide alleles may also play a role in this process, following a polygenic hypothesis (Höllinger et al. 2019). We investigated genome‐wide selective sweeps in three different populations: two previously studied (Wallberg et al. 2017) from the Mau region and Mt. Kenya, and a newly sequenced population collected recently from the Rwenzori Mountains on the Ugandan slope. We obtained transcriptomes from highland and lowland Ugandan honey bees and identified Differentially Expressed Genes (DEGs). Our research aims to address the following questions: (1) What are the specific genes within the r7 and r9 inversions that contribute to high‐altitude adaptation? (2) Are there other genomic regions that play a significant role in adaptation to high‐elevation habitats? (3) Could the two inversion polymorphisms influence gene expression within the inverted regions by maintaining divergent regulatory variants between haplotypes? (4) Do we find evidence for genetic divergence and differential gene expression associated with highland and lowland habitats? By answering these questions, we aim to better understand the genetic mechanisms underlying the adaptation of honey bees to diverse environmental conditions in East Africa.

## 2 Materials and Methods

### 2.1 Samples Collection and Molecular Methods

Honey bees were collected on Rwenzori mountains, Western Uganda. A total of six *A. m. monticola* colonies were sampled in the mountain forest area at an altitude ranging from 2100–3000 m a.s.l. inside the Rwenzori national park. Corresponding *scutellata* bees were collected in the nearby lowland savannah at an altitude of 1123 m a.s.l. from two beehives (Table S1). Returning forager honey bees were collected individually in a small glass vial and immediately killed by decapitation. The head was stored in RNA‐Later (Thermo Fisher) for RNA preservation, and the remaining body parts were stored in ultra‐pure ethanol (concentration > 99%) for DNA conservation. DNA was extracted from the full thorax from three bees per colony (highland: *n* = 18; lowland: *n* = 6) using the Puregene Tissue Kit (QIAGEN) following the manufacturer's procedure. Total RNA was extracted from 12 honey bee heads collected at different elevations: six were collected at high elevation and were sampled from two different colonies, while the remaining six were sampled in the lowland following the same sampling method. The manufacturer's protocol of the TRIzol reagent (Thermo Fisher) was used to perform the RNA extraction. Briefly, the honey bee head was homogenized in 500 μL of TRIzol, further processed using 100 μL chloroform, and precipitated with 300 μL isopropanol. After solvent evaporation, the RNA pellet was dissolved in 30 μL of RNase‐free water and stored at −80°C until further processing. The 24 samples were barcoded, and 2× 150 bp paired‐end reads were sequenced on an Illumina Novaseq 6000 platform targeting 25× coverage, an output of 10 Gb per sample for whole genome sequencing data, and 40 million reads with a total of 12 Gb output per sample for transcriptome sequencing (Biomarker Technologies (BMK) GmbH).

To get a more comprehensive picture of differentiation between the African honey bee populations, we integrated *monticola* and *scutellata* in a larger dataset including additional African honey bee subspecies from publicly available whole genome data (Short Read Archive (SRA; Table S2)). We included 11 *A. m. scutellata* (*n* = 11, 38× mean coverage) from Harpur et al. (2014; Bioproject: PRJNA216922); *A. m. adansonii* (*n* = 7), *A. m. capensis* (*n* = 12), *A. m. intermissa* (*n* = 15), *A. m. lamarckii* (*n* = 6), *A. m. monticola* (*n* = 6), and *A. m. scutellata* (*n* = 28), all with 68× mean coverage from Dogantzis et al. (2021; Bioproject: PRJNA729035); *A. m. monticola* (*n* = 1), *A. m. scutellata* (*n* = 5), and *A. m. jemenitica* (*n* = 3) all with 26× mean coverage from (Fuller et al. 2015; Bioproject: PRJNA237819). We included the dataset from Wallberg et al. (2017; Bioproject: PRJNA357367) comprising *A. m. scutellata* (*n* = 19) and *A. m. monticola* (*n* = 20) genomes, all with 12× mean coverage. Finally, we obtained new genome sequences of *A. m. monticola* (*n* = 18) and *A. m. scutellata* (*n* = 6) collected in the Rwenzori mountains and the surrounding savannah in Uganda, with a mean coverage of 25×.

### 2.2 DNA‐Seq Read Mapping and Variant Calling

Two datasets were generated. The first one included all honey bees described before. The second dataset included only the honey bee sequence data from (Wallberg et al. 2017) of Kenya and the newly sequenced honey bees from Uganda. Adapter sequences from raw Illumina sequencing reads were trimmed using cutadapt v2.6 (Martin 2011). For paired‐end reads, we additionally set the minimum read length ‘‐m’ to 50 and enabled the ‘paired‐output’ option for read trimming. The quality of the reads was assessed with fastqc v0.12.1 (Andrews et al. 2012). The reads were then aligned to the honey bee reference genome *Amel_HAv3.1* (Wallberg et al. 2019) using the burrow wheeler aligner (BWA) v0.7.17.1188 with the BWA‐MEM algorithm (Li and Durbin 2009).

SNP calling was carried out with ‘HaplotypeCaller’ from gatk4 v4.3.0.0, followed by joint genotyping using the ‘GenotypeGVCFs’ function (Poplin et al. 2017; Van der Auwera et al. 2013). Data were imported by chromosome with ‘GenomicsDBImport’ and a batch size of 20. The resulting VCFs were merged with bcftools v1.11 (Danecek et al. 2021). Allele balance for each variant was annotated with gatk3 v3.8.1.0 and filtered using vcffilter with the condition‘ABHet > 0.25 & ABHet < 0.75|ABHet < 0.01’, to allow for homozygous variants, too (Garrison et al. 2022).

After evaluating the distribution of the parameters from the INFO field in the final VCF, additional filters were applied with bcftools view v1.11 (Danecek et al. 2021). The filtering criteria for variant sites included the following thresholds: MQ < 40, MQRankSum < −5, MQRankSum > 5, ExcessHet > 20, AC = 1, INFO/DP < 11,660, INFO/DP > 21,654, AN < 826, FS > 20, SOR > 5, and QD < 5. Only biallelic SNPs were considered, and indels were excluded. The gatk4 function ‘SelectVariants’ was used to retain only variant sites in chromosomes for downstream analysis (Van der Auwera et al. 2013). Additionally, samples with a coverage of less than 6× were removed from the dataset. Finally, the remaining variants were phased using beagle v5.4 (22Jul22.46e.jar; Browning et al. 2018, 2021).

### 2.3 RNA‐Seq Data and Gene Expression Analysis

For transcriptome sequencing the reads quality was checked using fastqc (Andrews et al. 2012). Adapters were removed and reads were trimmed using trimmomatic v0.36 (Bolger et al. 2014). After trimming, fastqc was used again to check the quality of the trimming process. Trimmed reads were used by kallisto (Bray et al. 2016) to quantify the abundances of transcripts. Then, deseq2 (Love et al. 2014) was used to analyse differentially expressed genes. Finally, a GO enrichment analysis was done with gprofiler2 (Kolberg et al. 2023).

### 2.4 Principal Component Analysis Population Genetics Statistics and Inversion Classification

plink v1.90 (Chang et al. 2015) was used to convert the final .*vcf* file to .*bed*, .*bim*, and .*fam* plink format files. During the process, the dataset was filtered for Hardy–Weinberg equilibrium (‐‐hwe 0.0001), missing data (‐‐geno 0.01) and minor allele frequency (‐‐maf 0.01) and linkage disequilibrium, analyzing 50 k single nucleotide polymorphisms (SNP) at a time with a sliding window of 5 k SNPs and using a threshold of r2 of 0.5 (‐‐indep‐pairwise 50 5 0.5). Filtering pass SNPs were used to compute a principal component analysis (PCA) using the ‐‐pca plink function.

We calculated the population genetics parameters *F*_ST_, *D*xy, and *π* to further gain insights into the populations we are examining. The fixation index *F*_ST_ was used to quantify relative genetic differentiation between populations. *D*xy was calculated as the average number of nucleotide differences per site between populations, providing an estimate of absolute divergence between populations, whereas *π*, a measure of nucleotide diversity, is computed as differences per site within each population. All three of these indexes were computed using pixy v2.0.0 (Korunes and Samuk 2021). Also, where needed to test balancing selection, betascan2 was used as the main tool (Siewert and Voight 2020); this software allows us to compute beta values that are indicative of balancing selection. A value of 2 assigned by the tool could suggest balancing selection; higher and continuous values across a genomic region strengthen this assumption.

Previously identified r7 and r9 regions were then extracted from the final .vcf file using vcftools v0.1.16 (Danecek et al. 2011) with the information relative to breakpoints described in Christmas et al. (2018). As previously described, plink v1.90 was used to convert the dataset into plink file format applying the same filtering. Then, inversion polymorphisms in r7 and r9 were classified using the invclust R package. The *invClust* R package is designed to infer individual genotypes for chromosomal inversion using SNP. The method is based on a clustering‐based approach: (1) a dimensionality reduction is applied, then (2) INVCLUST applies a Gaussian mixture model to classify individuals into three distinct groups, and finally, (3) the clustering model is validated through Bayesian Information Criterion (BIC) (Cáceres and González 2015). This method was applied to Mau, Mt. Kenya, and Rwenzori populations.

### 2.5 McDonald‐Kreitman Test and Selection Scan (Selscan)

To compute the McDonald‐Kreitman test for genes within the r7 and r9 regions, the software degenotate (Mirchandani et al. 2024) was utilized. This test assesses the ratio of synonymous to non‐synonymous changes to identify genes potentially undergoing positive selection. However, the McDonald‐Kreitman test is limited by its exclusive focus on coding sequences and its tendency to be overly conservative. To address these limitations and to broaden the analysis, selscan v2.0 (Szpiech and Hernandez 2014) was additionally applied to identify genomic regions exhibiting significant deviations from neutrality. Within the framework of Selscan, the tool XP‐nSL (Szpiech et al. 2021) was used to detect signatures of selection in a test population given another population as reference. Highland versus lowland populations were compared using default parameters for the Mau region (highland: *n* = 9; lowland: *n* = 10), Mt. Kenya (highland: *n* = 10; lowland: *n* = 10), and the Rwenzori mountain system (highland: *n* = 18; lowland: *n* = 6). Raw XP‐nSL values were obtained (selscan flags ‐‐xpnsl ‐‐vcf mountain‐population.vcf ‐‐vcf‐ref savannah‐population.vcf) and scores were normalized with the companion program norm v1.3.0 (norm flags –xpnsl), considering everything above an XP‐nSL value of 2 or below −2 as potentially significant, as reported in the Selscan manual. During normalization, we also scanned by 10 kb non‐overlapping windows to detect regions of high differentiation. We then selected the top 1% positive scoring windows, which are those related to adaptation to high elevation habitat. The maximum value present in the window was assigned then to all genes within, as in (Szpiech et al. 2021). Positive values of the XP‐nSL statistic indicate a potential beneficial mutation in the test population, whereas negative values potentially indicate that the SNP is favorable in the reference population. By using this tool, we can get a more complete picture of the alleles involved in adaptation to high altitude in *Apis mellifera* .

Genomic positions above the threshold for each population were mapped to the latest version of the *Apis mellifera* .*gff* file using bedtools v2.26.0 (Quinlan and Hall 2010). Only SNPs mapping to a gene were kept, including the gene features promoter, untranslated regions, and coding sequences that were then used for a Gene Ontology (GO) enrichment analysis with gprofiler2 (Kolberg et al. 2023).

## 3 Results

### 3.1 Ugandan Honey Bees Are Highly Similar to Kenyan Honey Bees

**FIGURE 1:**
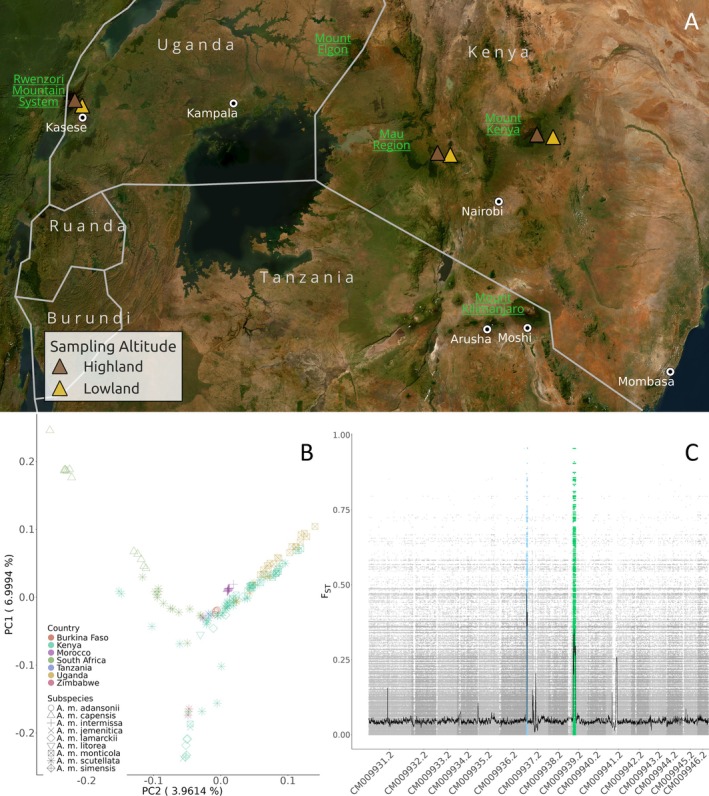
(A) Sampling location of the honey bees analyzed in this study, brown triangles represent honey bees collected at high elevation, while yellow triangles show honey bees collected in the nearby savannah. Ugandan honey bees were collected at an altitude ranging from 1123 m a.s.l. to 2600 m. Kenyan honey bees analyzed in this study were collected and described by Wallberg et al. (2017) (B) Principal component analysis of A‐Lineage honey bees from Africa. (C) Pairwise *F*_ST_ of highland (*n* = 18) and lowland honey bees (*n* = 6) population collected on Rwenzori mountain system, Uganda.

For the first time, we sampled honey bees inhabiting a distinct mountain system in western Uganda, known as the Rwenzori mountain system. This area, situated approximately 800 km from the previous sampling points, presented an intriguing research opportunity. A total of six honey bees were sequenced from the lowland savannah environment, and 18 were sequenced from the high mountain area. These honey bees were used along with those from Wallberg et al. (2017) to infer population genetics questions (Figure 1A).

After quality check and trimming, the dataset was left with 6,312,219 single‐nucleotide polymorphisms (SNPs) which were used for the following analysis. Whole genome SNPs were used to perform an explorative principal component analysis in order to observe if the newly sequenced Ugandan honey bees form a distinct cluster with respect to other honey bees belonging to the A‐Lineage (Figure 1B; Table S1). The PCA computed showed a clear distinction of some honey bee subspecies (i.e., *capensis, adansonii* and *intermissa*), while *monticola* and *scutellata* honey bees tend to cluster according to sample location (i.e., Kenya, South Africa) rather than subspecies (Figure 1B). This pattern holds also for newly sequenced samples for which it is not possible to observe a distinct cluster with respect to East African *monticola* and *scutellata* bees.

### 3.2 Inversion Polymorphisms in Uganda Mountain Honey Bees Are Substantially Different From Their Kenyan Counterparts

The following analysis was conducted using only the newly generated data and the Kenyan honey bees collected by Wallberg et al. (2017). This dataset is the only suitable one for our purposes since it includes honey bees collected in highland and lowland from geographically close populations (Figure 1A). Closer analysis of the populations considered revealed little to no differentiation between the three populations (Table 1) with the lowest *F*_ST_ value of ~0.002 found when considering Mau highland versus Mt. Kenya lowland and the highest value of ~0.028 found when considering Rwenzori lowland versus Mt. Kenya highland This pattern is consistent with both previous findings and the PCA which includes all samples (Figure 1B), where it is not possible to identify two clear clusters when considering the Rwenzori and Kenyan populations. Along with *F*_ST_, we also calculated Dxy and π. All together, these metrics can be used to assess diversity and differentiation since each one gives insight into something important that is not covered by the others (Figure S1; Table 1). Additionally, we ran Admixture with five different seeds with *K* = 1–6. The lowest cross‐validation error was detected at *K* = 1 for all five seeds considered (Table S3, Figure S2), supporting the view that all honey bees used in this study belong to the same ancestry.

**TABLE 1:** *Apis mellifera scutellata* and *Apis mellifera monticola* dataset and genetic differentiation (*F*_ST_).

Region	Population	Label	*N*	*π*	MF	MS	MKF	MKS	RWHL	RWLL
Mau	Highland	MF	9	0.1223	—	0.009	0.004	0.004	0.012	0.016
	Lowland	MS	10	0.1224	0.009	—	0.011	0.002	0.016	0.022
Mt. Kenya	Highland	MKF	10	0.1212	0.004	0.011	—	0.009	0.015	0.028
	Lowland	MKS	9	0.1216	0.004	0.002	0.009	—	0.017	0.023
Rwenzori	Highland	RWHL	18	0.1278	0.012	0.016	0.015	0.017	—	0.024
	Lowland	RWLL	6	0.1241	0.024	0.022	0.028	0.023	0.024	—

*Note:* The symmetric matrix represent the genome wide comparison between all population involved in this study. The low *F*_ST_ values highlight the very small genetic difference between highland and lowland honey bees in East Africa.

Abbreviations: MF, Mau forest; MKF, Mt. Kenya forest; MKS, Mt. Kenya savannah; MS, Mau savannah; RWHL, Rwenzori forest; RWLL, Rwenzori savannah.

As detected in previous studies, an *F*_ST_ significant increment was found in correspondence with previously described r7 and r9 regions, also when comparing Uganda highland honey bees to lowland Ugandan honey bees. This increase in *F*_ST_ values is evident despite the sample size of just six honey bees in the lowlands (Figure 1C).

Given the fact that population genetics statistics detected an increase in the genomic regions in correspondence of r7 and r9, an accurate classification of the r7 and r9 chromosomal inversion status was carried out using the *invClust* R package (Cáceres and González 2015). In the Rwenzori mountain population, the frequency of inverted homozygous individuals (INV) in r7 in the highland was 0.83, the heterozygous (HET) were 0.11, while homozygous inverted individuals were 0.06 (STD). In the lowland, no homozygous individuals were detected, while 0.5 were classified as heterozygous and 0.5 were classified as STD. In the r9 region in the highland, 0.83 honey bees were classified as INV and 0.17 were classified as HET. In the lowland, 0.2 individuals were assigned as HET, while the remaining 0.8 were classified as STD. In both situations, the increase of the inverted allele in the highland populations shows a clear association between highland environment and the INV genotype (Table 2).

**TABLE 2:** Frequency of r7 and r9 inversion polymorphisms in the three considered populations, and relative *F*_ST_ values when compared to other populations.

		Frequency		Genetic differentiation (*F*_ST_)					
				r9					
	Label	r7	r9	MF	MS	MKF	MKS	RWHL	RWLL
r7	MF	0.9	0.9	—	0.108	0.005	0.176	0.067	0.279
	MS	0.1	0.1	0.348	—	0.131	0.007	0.167	0.067
	MKF	1.0	0.89	0.045	0.480	—	0.199	0.073	0.242
	MKS	0.2	0.2	0.442	0.035	0.563	—	0.226	0.011
	RWHL	0.89	0.92	0.099	0.410	0.150	0.493	—	0.279
	RWLL	0.25	0.08	0.309	0.024	0.493	0.139	0.395	—

*Note:* The frequency reported is the frequency of the INV allele over the overall number of alleles; the lower triangle shows F_ST_ values relative to the r7 inversion; the upper triangle shows F_ST_ values relative to the r9 inversions.

Abbreviations: MF, Mau Forest; MKF, Mount Kenya Forest; MKS, Mount Kenya Savannah; MS, Mau Savannah; RWHL, Rwenzori Highland; RWLL, Rwenzori Lowland.

A similar inversion‐genotype classification was performed for populations from the Mau region and Mt. Kenya, using the same methodological framework as in Wallberg et al. (2017). In the Mau savannah, the INV, HET, and STD of the r7 inversion sample frequencies were 0.0, 0.3, and 0.7, respectively, while in the Mau forest environment, INV, HET, and STD were 0.89, 0.11, and 0.0. The r9 region follows a similar pattern, with two out of nine samples classified as HET in the highland and seven out of nine samples classified as INV. Lowland samples showed a decrease in the INV individuals, with two out of ten INV, two out of 10 HET, and the remaining ones classified as STD (Table 2).

The Mt. Kenya population followed a similar pattern. In the savannah r7, the STD allele was completely fixed, while in the highland, the INV allele reached fixation. The r9 region showed similar frequencies, with 0.9 INV and 0.1 HET individuals in the highland. In the Mt. Kenya savannah population, we could not observe INV samples, and the majority of collected specimens were classified as STD (0.78) with the remaining 0.12 being observed as HET (Table 2).

**FIGURE 2:**
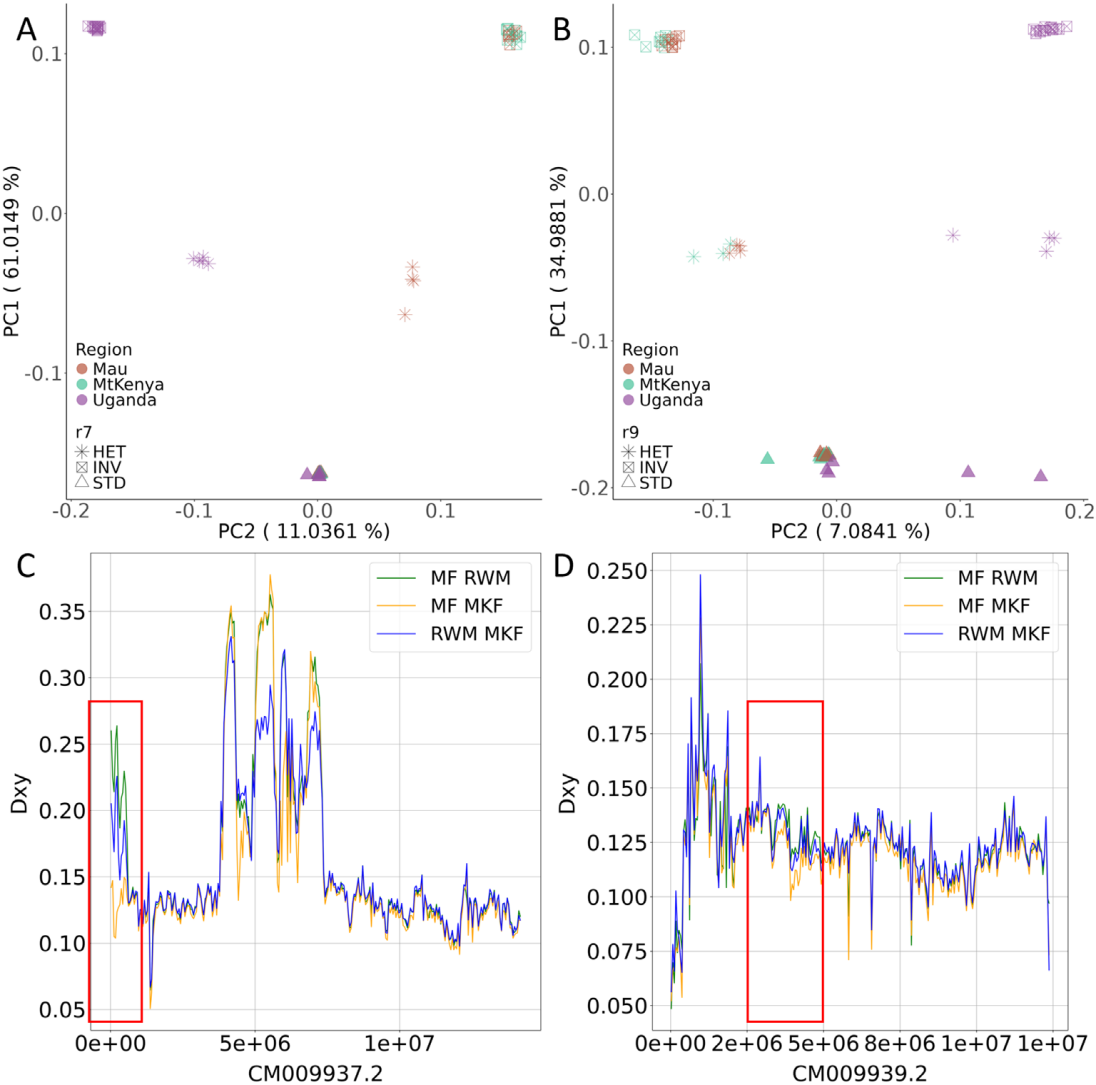
(A, B) Principal component analysis of r7 and r9 inversion polymorphisms, shape of the dots represents inversion status, while the color represents the population. (C, D) Sliding window Dxy, of highland population comparison. Populations show the same overall values except in correspondence of the inversion polymorphisms where comparison between Rwenzori mountain population (RWM) and Kenyan mountain population (MF and MKF) show a significant increase with respect to comparison between MF and MKF.

A PCA was computed for r7 and r9 regions only. The resulting plot clearly highlights different clusters relative to the genotype of the individuals (INV = inverted homozygous, HET = heterozygous, NI = non inverted homozygous). We observe that both the INV and HET samples are further subdivided between Kenyan and Ugandan samples (Figure 2A,B). This differentiation can be attributed to the presence of the inverted allele in both the INV and HET individuals. It is evident that the diversity is concentrated in this allele, as its absence is concomitant with the absence of the allele itself (i.e., NI honey bees in Figure 2A,B). The increased diversity derives probably from the lack of gene flow between the Ugandan inverted haplotype and Kenyan inverted haplotypes. We computed a sliding window Dxy on all chromosomes and found evidence of this differentiation in both chromosome 7 and chromosome 9. It is evident from Figure 2C,D that the average Dxy in r7 and r9 is larger for the comparison between the Rwenzori mountain highland population and highland Kenyan populations than for the comparison between highland Kenyan populations. Possibly the shift in frequency of many SNPs inside these regions is due to genetic drift and lack of gene flow between high mountain peaks in Uganda and Kenya; however, in all populations analyzed (Mau, Mt. Kenya and Uganda) the average Dxy observed is similar when comparing highland and lowland from the same mountain region. We also can observe that the average Dxy computed in r9 is relatively lower than the one observed in r7. This behavior could be attributed to the sizes of the two inversion polymorphisms, which may lead to differences in levels of recombination. The r7 region spans approximately 0.6 Mb, while the r9 region has an approximate dimension of 1.7 Mb. It is conceivable that genetic exchange may occur in inverted regions in the event of a double crossing‐over or gene conversion events (Rozas and Aguadé 1994; Schaeffer and Anderson 2005; Schaeffer et al. 2024). The probability of the first occurring, however, is thought to increase with the distance from the breakpoints (Navarro et al. 1997; Faria and Navarro 2010; Stevison et al. 2011). While we detected a considerable number of gene conversion tracts in both r7 and r9, which may represent the primary recombination force at work in these two inversion polymorphisms (data not shown), it is still probable that, given the size of the r9 region, double crossing‐over events occurred in the past, reducing haplotype difference even if the haplotype in Uganda significantly differs from the one observed in Kenya.

### 3.3 Chromosome 5 Shows r7‐ and r9‐Like Patterns

**FIGURE 3:**
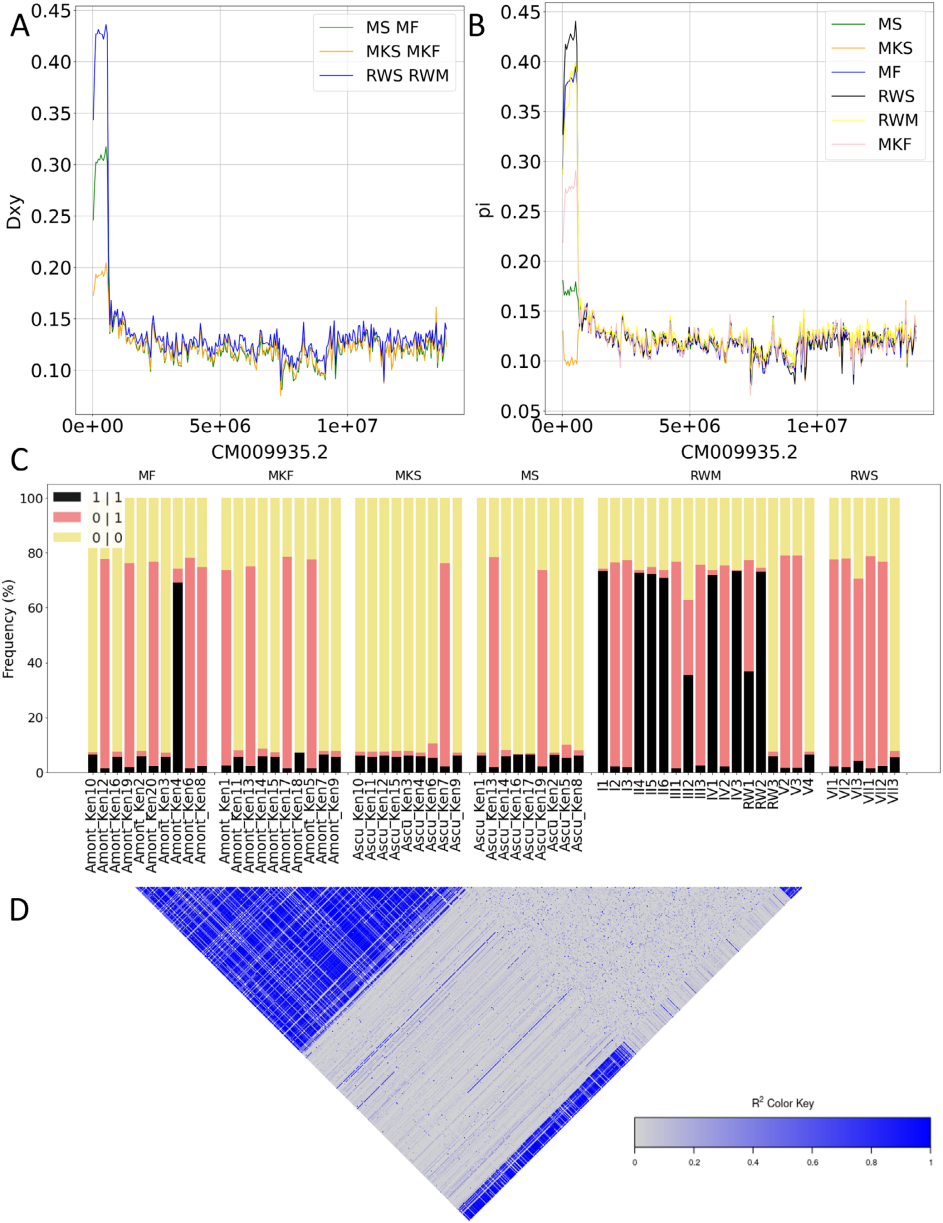
(A, B) Dxy and Pi sliding window of 10 kb non overlapping SNPs of chromosome 5. (C) Genotype distribution of homozygous alternative (1!1) heterozygous (0!1) and homozygous reference (0|0) variants inside the region. (D) Linkage disequilibrium pattern of the first half of chromosome 5, the LDheatmap R package was used to generate the heatmap (Shin et al. 2006).

We further explored the increased divergency detected at the beginning of chromosome five. The first SNP of increased Dxy value was detected at position 3811, with the last one at position 599,431 (Figure 3A,B). This makes this region ~0.6 Mb large. The region encompasses 64 genes; no specific enrichment was observed when a GO term analysis was performed with the 64 genes; however, we noted the gene PHRF1 (GeneID_727274) which is a PHD and ring finger domain 1 protein, and it is highly involved in the regulation of the epigenome by maintaining the correct DNA methylation patterns. We computed the genotype of single individuals, evaluating the quantity of 1|1 (homozygous alternative), 0|1 and 1|0 (heterozygous), and 0|0 (homozygous reference). We noted that the heterozygous individuals are mostly distributed across the different populations. In particular, the heterozygous genotype is present at a higher frequency in different populations (i.e., Uganda; Figure 3C). Interestingly, there are strong signs of LD (Figure 3D) supporting the view of potential recent selection signals given the high Dxy and nucleotide diversity, particularly in Rwenzori mountain samples. By using BetaScan2, we tested highland samples for signals of balancing selection given the very high amount of heterozygous sites in the newly discovered region. We observed long runs of heterozygosity with beta1 index values exceeding 2 in highland populations, indicating potential regions under balancing selection (Siewert and Voight 2020). In contrast, this pattern was not evident in lowland honey bee populations (Figure S3).However, it was difficult to confirm if this region actively involves structural variants.

### 3.4 Selection Scan Patterns Confirm r7 and r9 as Crucial to Highland Adaptation

A comparative analysis was conducted between highland and lowland populations utilizing XP‐nSL as the analytical tool. Along with this, we tested genes under positive/negative selection using the McDonald‐Kreitman test on genes in the r7 and r9 regions; however, no results were gathered with the exception of a few uncharacterized genes. This could be due to the nature of the test that considers only coding sequences and due to the sample size (Tables S4 and S5). The lowland population was employed as the reference group to identify evidence of adaptation to high elevation habitats. In all three cases, lowland savannah populations were kept as reference.

The genes falling within the top 1% differentiated windows were extracted and subjected to a GO enrichment analysis; the XP‐nSL value assigned to the window was then assigned to all genes that were falling inside the region as proposed in (Szpiech et al. 2021). The analyses were conducted against a database of annotated genes, with a threshold of significant GO terms offset at 0.05 and applying the g:SCS correction for multiple testing (Kolberg et al. 2023). Our analysis revealed a variable number of genes involved in each population. In Rwenzori mountains population XP‐nSl a total of 302 genes were detected, in the Mau region from the selscan analysis, and after a GO term analysis, no results could be gathered. The Mt. Kenya yielded the most abundant result (494 genes), however, as for the Mau region, no significant GO terms were observed. Finally, the analysis performed on the Rwenzori mountain population exhibited a total of 262 genes; the following analysis revealed GO terms involved in cognition, memory formation, and G‐protein coupled receptor activity (Tables S6–S9). A truly remarkable note is that the highest XP‐nSL values were observed in the r7 region in all three populations (Figure S4), with octopamine receptors consistently ranking among the top values. A comparable pattern can be appreciated in the r9 region; although the values reported here are not as high as those located in r7. This outcome was anticipated in light of the genetic differentiation between the two regions and the previously calculated *F*_ST_ and Dxy values. Finally, we could not observe the same pattern for the region of increased diversity observed at the beginning of chromosome five.

### 3.5 Differentially Expressed Genes Are Enriched for GO Terms Involved in Stress Adaptation

**FIGURE 4:**
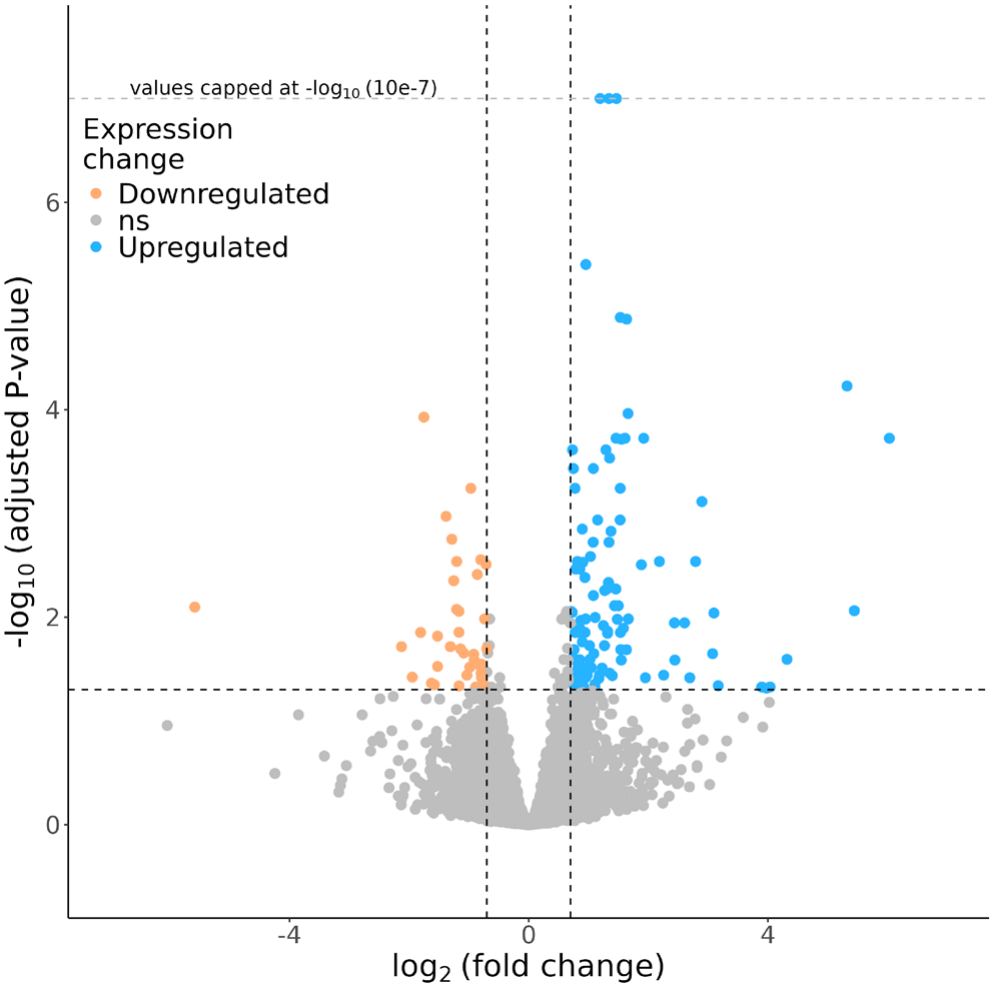
Volcano plot of Differentially Expressed Genes (DEGs), blue and orange dots represent up‐ and downregulated genes in the highland respectively. Significance level was capped at 10e‐7 for a better representation of data. A total of 39 genes were detected as downregulated (orange) and a total 113 were detected as upregulated (blue), given a threshold of log_2_FC = ±0.7.

We enriched our dataset by 12 transcriptomes obtained for the first time from honey bees of the Rwenzori mountains. Following the removal of low‐quality reads, the lowland samples which served as control cases had an average of approximately 40 million reads and a GC content of 45%, whereas the highland honey bees had an average of approximately 41 million reads and a GC content of 46%. A total of 181 DEGs were identified with an adjusted *p*‐value lower than 0.05. However, to reduce the discovery of false positives, we selected a threshold of log_2_ fold change (log_2_FC) of ±0.7. In total, 29 genes were cut out from the analysis, leaving the dataset with 152 effective DEGs, divided into 39 downregulated genes and 113 exhibiting increased expression (Figure 4). A GO enrichment analysis was conducted on up‐and downregulated genes independently against the annotated gene set of *Apis mellifera*, with a *p*‐value threshold of 0.05 and the g:SCS correction for multiple testing (Kolberg et al. 2023). When analyzing only the downregulated genes, no significant GO terms were observed, while when we considered only upregulated genes, we found evidence of 33 different GO terms involved in total. Seven of these were identified in the context of molecular function (MF), 21 in the context of biological pathways (BP) and five enriched KEGG pathways (Table 3).

**TABLE 3:** GO enrichment analysis using DEGs.

GO ID	Term name	Source	*p*
GO:0016491	Oxidoreductase activity	MF	3.83E‐04
GO:0102965	Alcohol‐forming long‐chain fatty acyl‐CoA reductase activity	MF	3.92E‐03
GO:0016620	Oxidoreductase activity, acting on the aldehyde or oxo group of donors, NAD or NADP as acceptor	MF	1.10E‐02
GO:0016885	Ligase activity, forming carbon–carbon bonds	MF	1.87E‐02
GO:0080019	Alcohol‐forming very long‐chain fatty acyl‐CoA reductase activity	MF	2.28E‐02
GO:0003824	Catalytic activity	MF	2.53E‐02
GO:0016903	Oxidoreductase activity, acting on the aldehyde or oxo group of donors	MF	4.09E‐02
GO:0044281	Small‐molecule metabolic process	BP	1.23E‐05
GO:0043436	Oxoacid metabolic process	BP	7.70E‐05
GO:0019752	Carboxylic acid metabolic process	BP	7.70E‐05
GO:0006082	Organic acid metabolic process	BP	8.29E‐05
GO:0032787	Monocarboxylic acid metabolic process	BP	1.20E‐04
GO:0044283	Small‐molecule biosynthetic process	BP	1.49E‐04
GO:0006739	NADP metabolic process	BP	1.50E‐04
GO:0006633	Fatty acid biosynthetic process	BP	2.05E‐04
GO:0072330	Monocarboxylic acid biosynthetic process	BP	2.05E‐04
GO:0046394	Carboxylic acid biosynthetic process	BP	7.87E‐04
GO:0016053	Organic acid biosynthetic process	BP	9.04E‐04
GO:0006631	Fatty acid metabolic process	BP	1.10E‐03
GO:0006629	Lipid metabolic process	BP	2.14E‐03
GO:1901568	Fatty acid derivative metabolic process	BP	2.38E‐03
GO:0072524	Pyridine‐containing compound metabolic process	BP	2.75E‐03
GO:0006098	Pentose‐phosphate shunt	BP	4.74E‐03
GO:0051156	Glucose 6‐phosphate metabolic process	BP	4.74E‐03
GO:0006740	NADPH regeneration	BP	4.74E‐03
GO:0005975	Carbohydrate metabolic process	BP	1.15E‐02
GO:0046496	Nicotinamide nucleotide metabolic process	BP	3.80E‐02
GO:0019362	Pyridine nucleotide metabolic process	BP	3.80E‐02
KEGG:01100	Metabolic pathways	KEGG	5.81E‐04
KEGG:01212	Fatty acid metabolism	KEGG	4.89E‐03
KEGG:01200	Carbon metabolism	KEGG	9.49E‐03
KEGG:00030	Pentose phosphate pathway	KEGG	1.93E‐02
KEGG:04146	Peroxisome	KEGG	1.95E‐02

*Note:* The threshold selected for significant genes to be submitted to the GO term analysis was ±0.7 log_2_FC and *p*‐value ≤ 0.05.

Abbreviations: BP, biological process; CC, cellular component; KEGG, Kyoto Encyclopedia of Genes and Genomes; MF, molecular function.

### 3.6 r7 and r9 Inversion Polymorphisms Likely Alter Gene Expression

**FIGURE 5:**
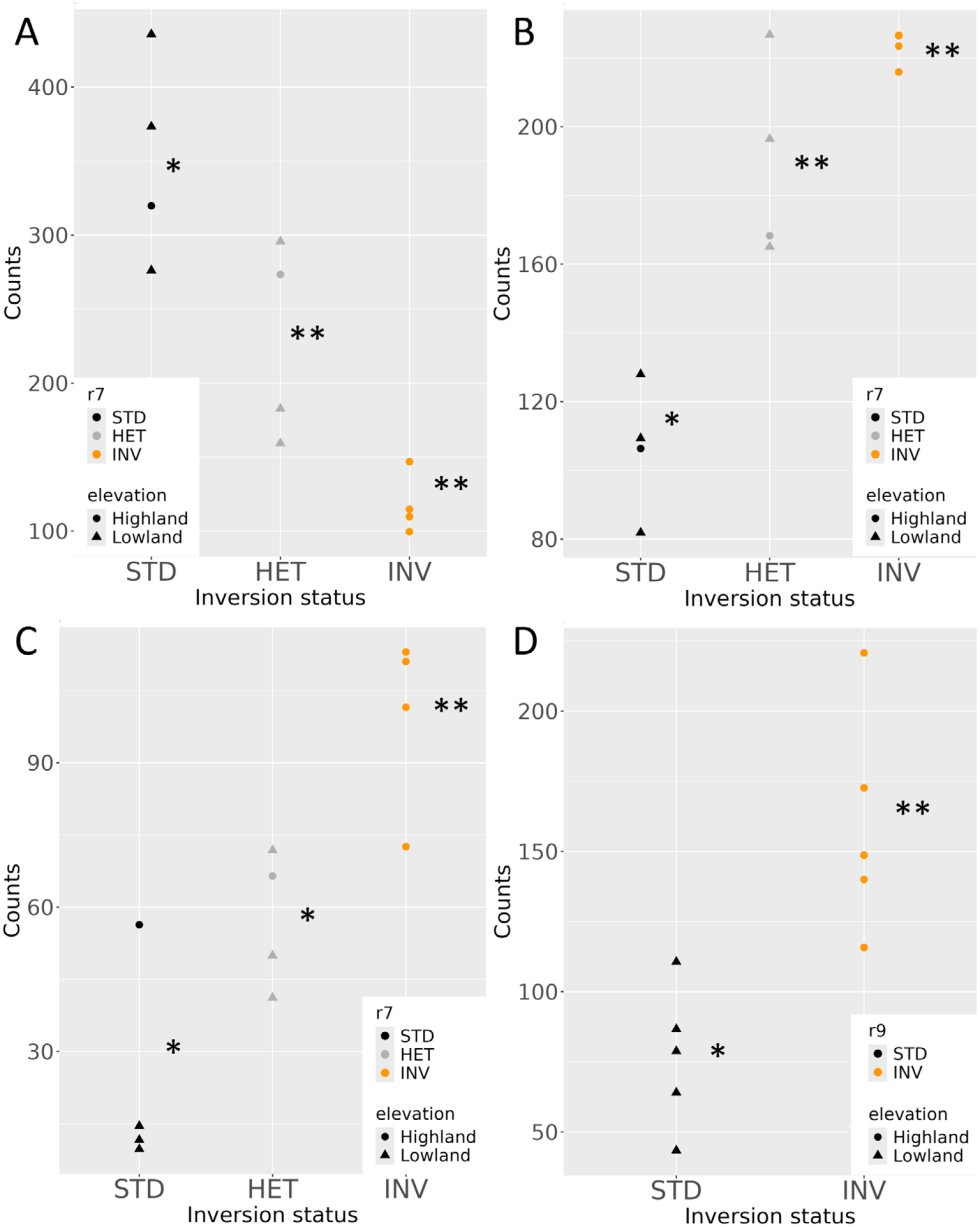
Differential expression in different r7 and r9 inversion genotypes (STD= non‐inverted homozygous, HET = heterozygous, INV = inverted homozygous). Expression patterns of LOC413698 (A), LOC552586 (B), LOC552005 (C), located in the r7 inversion, and LOC409025 (D), located in the r9 inversion. This gene has only two inversion statuses due to the lack of HET individuals in the population. Colors define the inversion status, while shape defines the sampling elevation. Groups of samples flanked by the same number of stars are not statistically different given a Tukey's HSD test.

To further test the effect of the r7 and r9 inversion polymorphisms on the gene expression, we divided samples based on inversion status rather than elevation. This left the dataset with four INV, four HET and four STD samples. We then analysed the expression levels of genes in the three different status the r7 inversion status as variable in the DESeq2 model. This was done to better understand the expression patterns of STD, INV and HET all together. DESeq2 yielded a total of four DEGs when STD was placed against INV with three of them (LOC413698, LOC552586, LOC552005) residing inside the r7 region (Figure 5). While a comparison between STD and HET, and HET and INV did not yield any significant results regarding genes inside the r7 inversion. The expression levels of the three detected genes were plotted to see how the expression of RNA changes when inverted alleles are present (INV and HET). In case of LOC413698, HET and STD samples are not statistically different between each other, but they are significantly different from INV samples. In the case of LOC552586, HET samples have higher expression rates than STD individuals and are significantly different from them. INV samples show a similar pattern. Finally, in LOC552005 STD and HET samples are not statistically different, but both are statistically different from INV samples. It is truly remarkable that data from all three cases demonstrate a correlation between the presence or absence of the inverted allele and the expression rates of these three genes (Figure 5; Table 4).

**TABLE 4:** Significant differentially expressed genes (DEGs) with a model accounting for inversion status only.

Gene	Log_2_fc	*p* (DESeq2)	Comparison	*p* (Tukey HSD)
LOC413698			STD – INV	6.58e‐04**
	−1.582	7.50e‐05**	STD – HET	3.19e‐02*
			HET – INV	5.38e‐02
LOC552586			STD – INV	4.75e‐05**
	1.074	9.35e‐03*	STD – HET	6.57e‐04**
			HET – INV	9.31e‐02
LOC552005			STD – INV	6.89e‐04**
	2.263	1.19e‐02*	STD – HET	6.79e‐02
			HET – INV	2.68e‐02*
LOC409025			N/A	N/A
	1.051	4.67e‐02*	N/A	N/A
			N/A	N/A

*Note:* Tukey's Honestly Significance Difference (HSD) test was applied to understand weather all three groups, inverted homozygous (INV), non‐inverted homozygous (STD), and heterozygous (HET), where equal or different. A star (*) near the *p*‐value represent a significance level of *α* ≤ 0.05, two stars near the *p*‐value represent a significance level of *α* ≤ 0.001. N/A indicates that the comparison could not be performed due to the absence of HET individuals; therefore, only the DESeq2 analysis is reported for these cases.

The same comparison involving STD, HET, and INV haplotypes could not be performed on r9 due to a lack of heterozygous specimens, and only a comparison of STD against INV could be made. In this case, only LOC409025 (apolipoprotein D, log_2_FC = −1.051, *p* = 4.61e‐02) was found to be significant; however, this is a very important result since it shows that r9 could potentially alter gene expression (Figure 5; Table 4).

A comparison was conducted between the results of the selection scan and the DEGs to identify commonalities. This revealed that five genes were shared between the two datasets. LOC551123 is an RNA‐binding protein Musashi which in *Drosophila* positively regulates neural differentiation and negatively regulates the hypoxia‐inducible factor (HIF) pathway. Furthermore, it is involved in the determination of cell fate and the cellular response during normoxic and hypoxic conditions (Bertolin et al. 2016; Landínez‐Macías and Urwyler 2021). LOC413698 is the octopamine receptor beta‐1R which is involved in behavior and social division of labor in honey bees (Balfanz et al. 2013; Rein et al. 2013; Reim and Scheiner 2014; Schilcher et al. 2021). Furthermore, octopamine in honey bees is responsible for elevating appetite levels when the organism is hungry (Akülkü et al. 2021). LOC408343 encodes a potassium voltage‐gated channel (Shaker protein) which in PC12 cells is involved in hypoxia sensitivity. Finally, LOC552844 encodes a complexin which is a SNARE protein responsible for the regulation of neurotransmission (Cho et al. 2014). Among the previously described genes, LOC413698 is the only one located in the r7 region.

## 4 Discussion

The adaptation of *Apis mellifera* to high altitude encompasses a multitude of parameters. The high mountain environment presents a dual challenge. On one hand, certain behavioral adaptations are required, while on the other hand, different selection regimes are needed for specific genes in highland and lowland environments to enable the organisms to cope with it, such as tolerance to hypoxia or pathogens. The sequencing of 24 new honey bees collected on the Ugandan slope of Rwenzori Mountains highlights the presence of r7 and r9, two previously described inversion polymorphisms (Wallberg et al. 2017; Christmas et al. 2018), which display high frequency in highland populations. This finding suggests that these polymorphisms could be present on other East African mountain systems that have not yet been studied. Moreover, evidence indicates that *A.m. monticola* honey bees collected on Rwenzori Mountains r7 and r9 regions genetically drifted from those collected in Kenya. The frequency of the inversions in the highland suggests, however, that both r7 and r9 capture adaptive alleles for highland adaptation. The low gene flow and genetic drift contributed to shaping the genetic diversity between the two ~800 km away sampling areas. An *F*_ST_ scan conducted using only INV individuals from the Rwenzori Mountains population and Kenyan populations revealed a high number of highly differentiated SNPs; this was confirmed also through a sliding window Dxy (Figure 2A,B). In many instances, these mutations are fixed in one of the populations and are absent or present at very low frequency in the other.

We conducted an in‐depth analysis of the newly described region on chromosome 5, and found a high level of differentiation (Dxy) between highland and lowland populations. Nucleotide diversity (*π*) was generally low in lowland populations, with the exception of RWLL. However, this exception may be attributable to sample size limitations (Figure 3A,B). We could also observe high levels of heterozygosity and LD in the region, suggesting the potential involvement of a structural variant. However, we have not been able to define it. Finally, given the high level of heterozygosity in the highland population, we tested for balancing selection, and we found evidence for it in the highland population (Figure S3), which is not evident in the lowland, suggesting that the heterozygous individuals can benefit from it in highland environments. This result could benefit from additional data from more populations, including an increased sample size.

The selection scan analysis proved inadequate in detecting significant and crucial GO terms in the Mau and Mt. Kenya regions, with only a few terms observed in the Rwenzori mountain population, predominantly involved in G‐protein coupled receptor activity, memory, learning, and cognition (Table S7). An exceptional finding is that r7 and r9 were consistently detected in the analysis of the three populations. An eye‐catching result is that Octopamine receptors were ranked by the algorithm among the genes with the highest XP‐nSL values in all three populations, supporting the view of one central likely candidate of selection in highland environments. Octopamine receptors are G‐protein coupled receptors and were already pointed out as a potential candidate for adaptation to high elevation (Wallberg et al. 2017). Their role has been extensively studied in honey bees in relation to a range of behavioral traits, including division of labor, appetite regulation, memory formation, learning, and thermogenesis (Kaya‐Zeeb et al. 2022). Although octopamine, the neurotransmitter associated with these receptors, plays a crucial role in these behaviors, a clear association with adaptation to high elevation in honey bees has yet to be established. Studies on *Locusta migratoria* suggested that octopamine is important for the correct transmission of signals during hypoxic conditions (Money et al. 2016).

Despite the detection of genes that could play a fundamental role in adaptation to high elevation such as G protein‐coupled receptors, which are also involved in hypoxia tolerance in certain human tissues, no direct connection was found between our results and the three main genes involved in the hypoxia initiation pathway (Azevedo et al. 2011). AmSima (LOC408852), AmTango (LOC411234) and AmFatiga (LOC413929).

RNA‐seq was employed to ascertain the preferred pathways of mountain bees in comparison to lowland savannah honey bees. In total, 12 individual transcriptomes were compared; 29 downregulated and 113 upregulated genes were detected with an adjusted *p*‐value of 0.05 and a log_2_FC of ±0.7. The GO terms analysis of downregulated genes did not reveal any significant GO term, despite the presence of one of the previously suspected candidate genes (LOC413698, octopamine receptor beta 1). On the other hand, the GO term analysis on upregulated genes revealed an enrichment of different terms that were already associated with hypoxic environments. Notably, the enrichment for oxidoreductase activity (which includes oxidoreductase activity acting on the aldehyde or oxo group of donors, NAD or NADP as acceptor) and the carbohydrate metabolic process were detected in *Tribolium castaneum* when exposed to very low oxygen concentration (Wang et al. 2018). In particular, numerous genes involved in the carbohydrate pathway were identified, the majority of which were associated with glycolysis. All these genes exhibited increased expression in the highland population, suggesting that these proteins may play a pivotal role in optimizing energy consumption in non‐normoxic conditions. We found log_2_FC differences in proteins involved in the carbohydrate pathway ranging from 0.43–0.55 (LOC551103: probable pyruvate dehydrogenase, Pyruvate Kinase: LOC552007 and Enolase: LOC552678) to 1.58 (LOC411202: 1,5‐anhydro‐D‐fructose reductase). We hypothesize that honey bees analyzed in this dataset are not experiencing extreme hypoxia; however, they could be adapted to lower levels of oxygen with respect to honey bees observed in the lowland savannah. Many GO terms we observed are associated in some way with response to a lower level of oxygen availability. We suggest that the adaptation observed among the highland population involves an increased expression of such pathways, albeit not to the same extent as in other populations or laboratory experiments (Wang et al. 2018). Other notable genes detected include Hsp70‐4, which is upregulated in highland honey bees. This gene encodes for a heat shock protein cognate 4, which is involved in low oxygen tolerance in numerous species (Wen et al. 2021) and longevity (KEGG: ame04213); and LOC411569, which encodes for a TANC2‐like protein. A distinguished role of this protein is that it functions as a signal for the inhibition of the mTOR pathway in mammals (Kim et al. 2021). The mTOR pathway inhibition is caused by hypoxia, and the gene encoding for mTOR in honey bees has been found to be under selection in highland Africanized bee populations (Everitt et al. 2023). In the present study, it was not possible to ascertain a definitive involvement of hypoxia‐related factors in this context. This is likely due to the absence of extreme hypoxia conditions; however, an adaptation to high elevation habitats was clear through the upregulation of genes involved in pathways aimed at mitigating the effects of low oxygen levels. A significant proportion of studies where very high expression levels of hypoxia factors are observed involve organisms exposed to very low oxygen concentrations (Azevedo et al. 2011; Wang et al. 2018).

Interestingly, we observed an enrichment of DEGs in KEGG:04146, representing the peroxisomes pathway. Peroxisomes are intracellular organelles involved in a multitude of metabolic processes such as fatty acid oxidation, lipid synthesis, and reactive oxygen species (ROS) metabolism. Additionally, they serve as a barrier against hypoxic stress (Jain et al. 2020; He et al. 2021).

Finally, we identified in DEGs some genes associated with the cuticular hydrocarbons (CHC) pathway. CHCs are vital molecules for honey bees, facilitating communication and preventing desiccation. They help honey bees recognize one another, and to a certain extent, they are specific to each hive. Distinct patterns of CHC exist in multiple species. High altitude natal fruit flies ( *Ceratitis rosa* ) exhibit markedly disparate CHC profiles between highland and lowland populations; the same is valid for Himalayan bumble bees, which have different CHC compositions based on elevational gradient (Vaníčková et al. 2015; Narah et al. 2024). Our analysis identified a notable number of genes with differentially expressed patterns implicated in the CHC pathway. LOC412166 is a fatty acyl‐CoA reductase involved in the CHC pathway. We also observed LOC412166 and LOC552417, which both encode for an acyl‐CoA Delta(11) desaturase. LOC725031 encodes for an elongation protein; these classes of proteins are crucial to determine the length of the fatty acid that will compose the final CHC profile. LOC412815 and FAR1 encode for a fatty acid synthase and a fatty acyl‐CoA reductase 1 and are central genes in the formation of CHCs.

Following a thorough analysis, no evidence was found to indicate different gene expression patterns within the r7 and r9 inversion polymorphisms, except for a few genes. Two genes were significantly downregulated: LOC413698 (Octopamine receptor beta 1 log_2_FC = −1.03, *p* = 0.03) and LOC726656 (Ca2+ independent phospholipase A2 gamma log_2_FC = −0.63, *p* = 0.04). Regarding r9, no gene was identified as significantly differentially expressed. Subsequently, an in‐depth investigation was conducted to ascertain the impact of r7 and r9 inversion on gene expression. The data were analyzed using the r7 inversion status as variable in the DESeq2 model which lead to the detection of expression differences in three genes when STD and INV were compared LOC413698 (octopamine receptor beta 1 log_2_FC = −1.582, *p* = 7.50e‐05), LOC552586 (cytochrome oxidase assembly factor 7 homolog log_2_FC = 1.074, *p* = 9.0e‐03) and LOC552005 (uncharacterized log_2_FC = 1.017, *p* = 1.19e‐02). For r9, we were only able to compare INV and STD individuals due to a lack of sufficient HET samples. Given the evident limitation we could however extract a very interesting result, in which we could observe LOC409025 as differentially expressed (log_2_FC = −1.051, *p* = 4.61e‐02). This gene encodes for a Apolipoprotein D, which was already described as protein involved in stress response in insects (Zhou et al. 2020). Together with the other genes could be a target for adaptation mechanism to high elevational habitats. However, this result may be subject to bias due to the potential influence of environmental factors acting directly on gene expression. The extent of this possible bias needs further exploration to understand its impact on our findings. To gain a more comprehensive understanding of the genes whose expression levels are altered by these factors, it is essential to extend the sampling and sequencing of INV, HET and STD individuals from a single elevation, thereby reducing the impact of environmental variables. Together with the detection of the r7 and r9 inversions and the RNA‐seq study performed here in this study we could clearly observe divergent selection acting in highland environments in the two inversion polymorphism. Nevertheless, it remains unclear whether *A. m. monticola* represents genuine adaptations to high‐elevation habitats or if their ability to survive in such environments is due to the high degree of plasticity exhibited by *Apis mellifera* . Addressing this question is challenging but crucial. Future studies should consider translocation and gene expression experiments, relocating lowland *A. m. scutellata* bees to the highland mountain forest environment and highland *A. m. monticola* bees to the lowland savannah environment. Additionally, evaluating the distribution and frequency of r7 and r9 in other East African mountain systems (e.g., Mt. Kilimanjaro) will help determine the significance of these chromosomal rearrangements and the genes they encompass. Such studies will not only enhance our understanding of bee adaptation but also inform conservation strategies in the face of environmental changes.

## Author Contributions

**Victor Sebastian Scharnhorst:** resources (supporting), writing – review and editing (supporting). **Ricarda Scheiner:** conceptualization (lead), funding acquisition (lead), project administration (lead), supervision (lead), writing – review and editing (equal). **Martin Hasselmann:** conceptualization (lead), funding acquisition (lead), project administration (lead), supervision (lead), writing – original draft (equal), writing – review and editing (equal). **Florian Loidolt:** data curation (supporting), writing – review and editing (equal). **Philipp Meyer:** resources (supporting), writing – review and editing (supporting). **Sonja Kersten:** data curation (equal), resources (equal), software (equal), writing – review and editing (supporting). **Patrick Vudriko:** writing – review and editing (supporting). **Deborah Ruth Amulen:** writing – review and editing (supporting). **Marco Mazzoni:** data curation (equal), formal analysis (lead), visualization (lead), writing – original draft (lead), writing – review and editing (lead).

## Conflicts of Interest

The authors declare no conflicts of interest.

## Supporting information


**Figure S1:** Genome wide and pi scan of analyzed population using a sliding window of 10 kb.

**Figure S2:** Admixture cross‐validation error from *K* = 1 to *K* = 6 for five different random seeds.

**Figure S3:** Beta‐values computed for highland and lowland populations with a set threshold of 2 using a 150 SNP window.

**Figure S4:** Selection scan XP‐nSL values for the three populations analyzed: Mau (Top), Mt.Kenya (Center) and Rwenzori Mountains (Bottom). The black line shows sliding window values of 10 kb. Red dots represent potential SNPs involved in high elevation adaptation, while blue dots represent potential SNPs involved in lowland adaptation.

**Table S1:** Uganda elevation collection and coordinates.
**Table S2:** ece371846‐sup‐0005‐Tables.xlsx. Samples included in the study and relatives coordinates of collection (where it was described in the original paper).
**Table S3:** ece371846‐sup‐0005‐Tables.xlsx. Admixture cross‐validation results for 5 different random seeds for K going from 1 to 6.
**Table S4:** ece371846‐sup‐0005‐Tables.xlsx. MKT results for genes inside the r7 inverted region.
**Table S5:** ece371846‐sup‐0005‐Tables.xlsx. MKT results for genes inside the r9 inverted region.
**Table S6:** ece371846‐sup‐0005‐Tables.xlsx. Genes falling inside 1% windows in Mau region.
**Table S7:** ece371846‐sup‐0005‐Tables.xlsx. Genes falling inside 1% windows in Mt. Kenya region.
**Table S8:** ece371846‐sup‐0005‐Tables.xlsx. Genes falling inside 1% windows in Rwenzori mountain region.
**Table S9:** ece371846‐sup‐0005‐Tables.xlsx. GO terms for selection scan analysis of Rwenzori region population.

## Data Availability

Illumina WGS reads of the newly sequenced 24 honey bees as well as RNASeq reads of the 12 honey bees used in this study are available at the NCBI SRA under the accession no. PRJNA1179698. The Figures [Link], [Link] and Tables S1–S9 as well as the scripts used to perform the analysis and plot the graphs are available at https://github.com/MarcoMazzoni93/Uganda.
