# Supplementary figures and images for: Genomic Landscape of High‐Altitude Adaptation in East African Mountain Honey Bees ( Apis mellifera )
Source: Ecol Evol. 2025 Aug 20;15(8):e71846. doi: 10.1002/ece3.71846 (PMC12367272; doi:10.1002/ece3.71846)

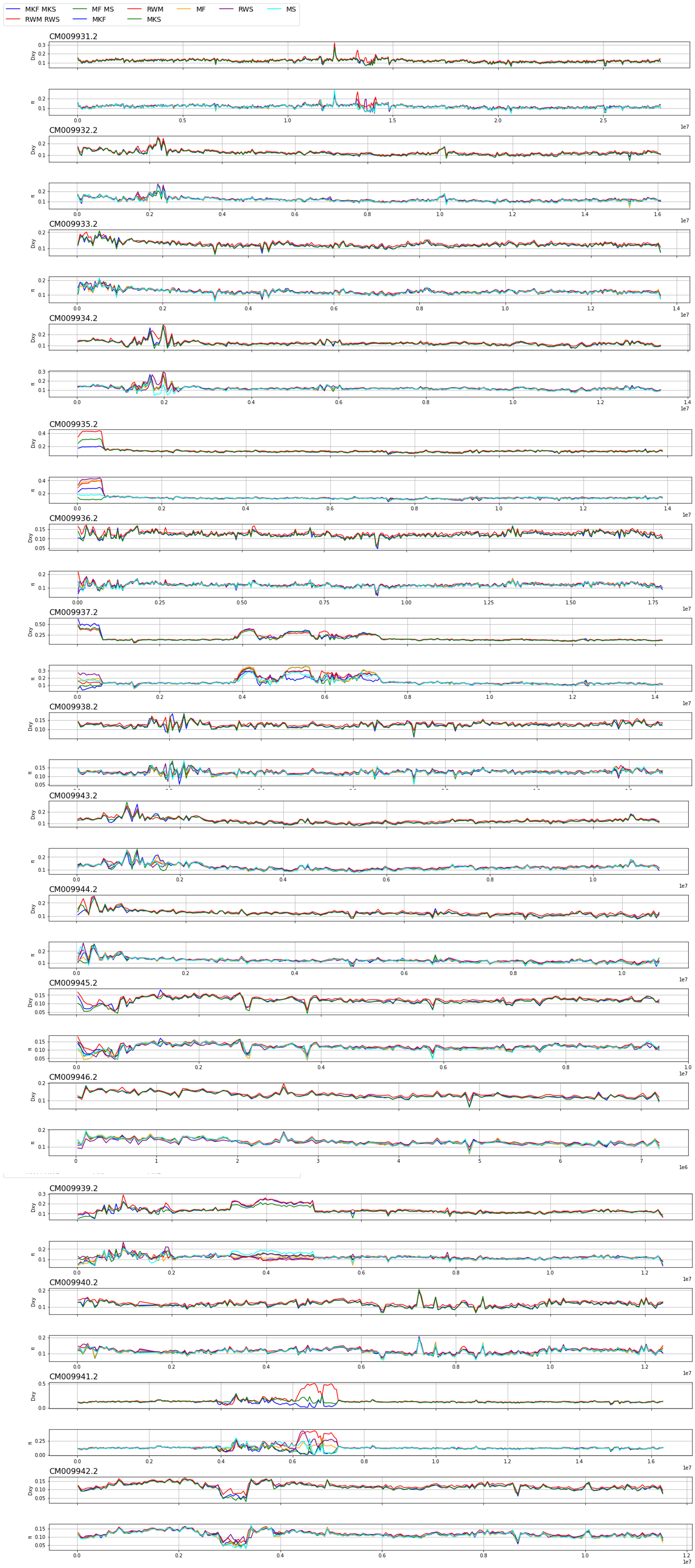

Supplement: Supplementary file 1 — Figure S1: Genome wide and pi scan of analyzed population using a sliding window of 10 kb. [file ECE3-15-e71846-s003.png]

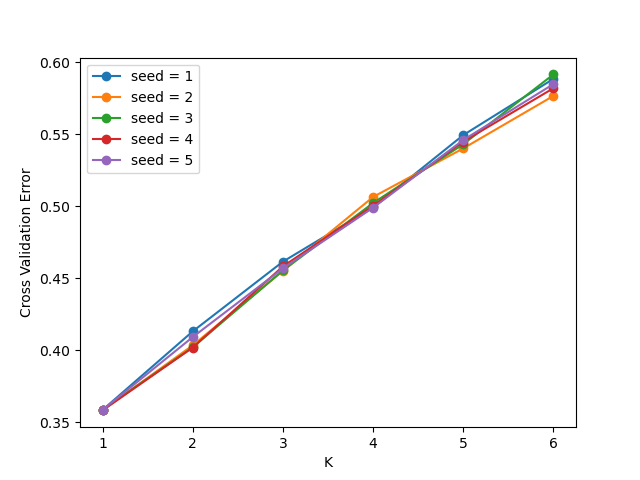

Supplement: Supplementary file 2 — Figure S2: Admixture cross‐validation error from K = 1 to K = 6 for five different random seeds. [file ECE3-15-e71846-s004.png]

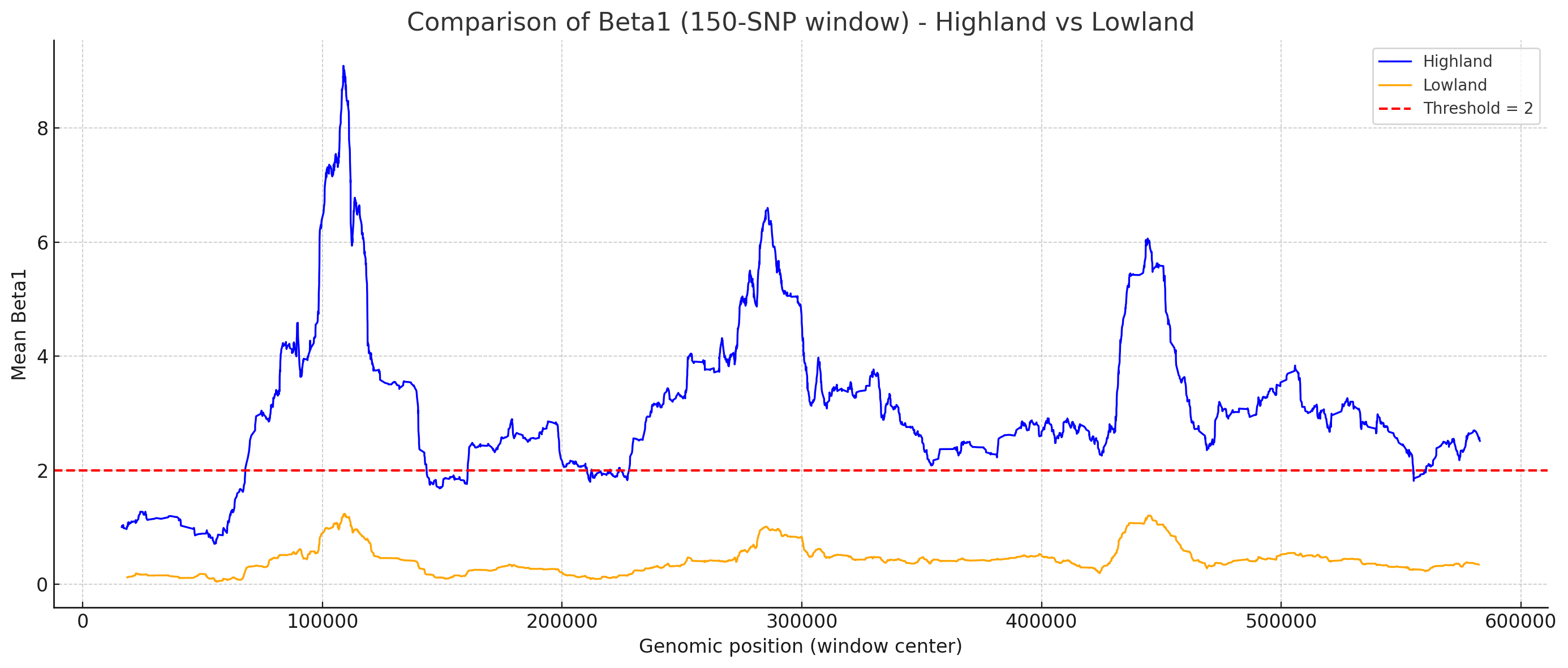

Supplement: Supplementary file 3 — Figure S3: Beta‐values computed for highland and lowland populations with a set threshold of 2 using a 150 SNP window. [file ECE3-15-e71846-s001.png]

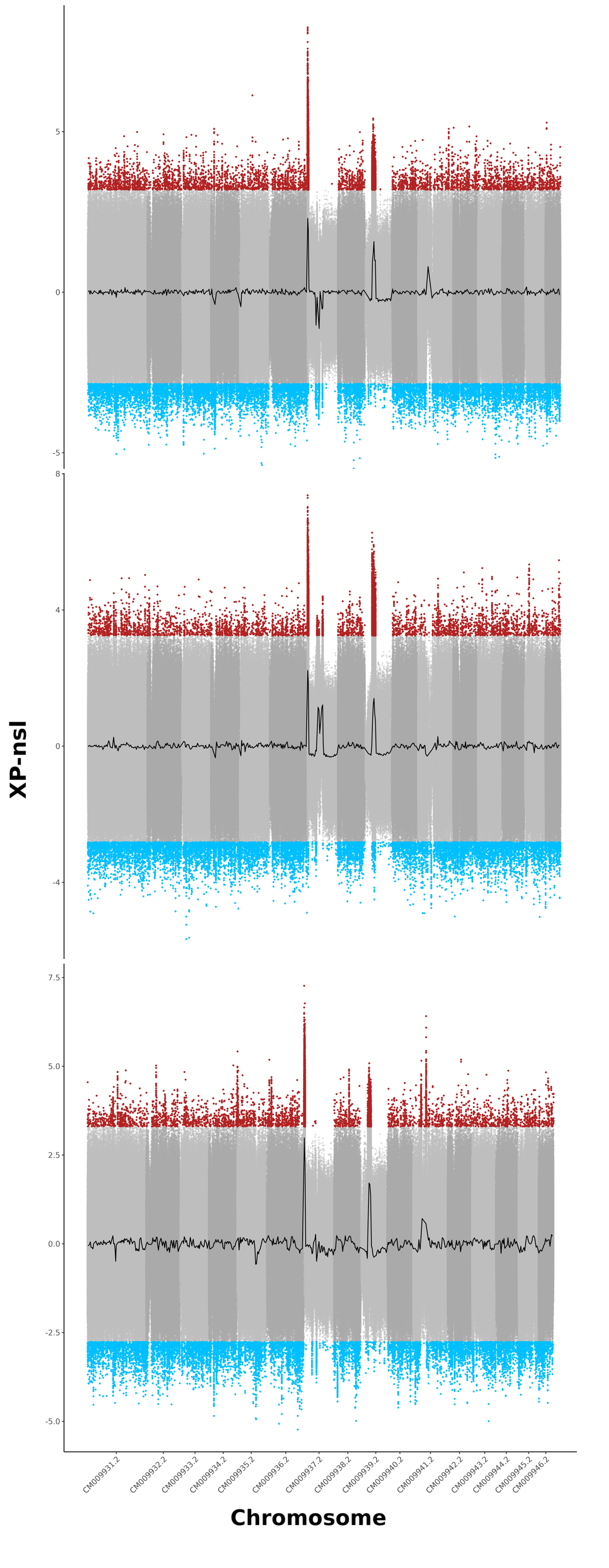

Supplement: Supplementary file 4 — Figure S4: Selection scan XP‐nSL values for the three populations analyzed: Mau (Top), Mt.Kenya (Center) and Rwenzori Mountains (Bottom). The black line shows sliding window values of 10 kb. Red dots represent potential SNPs involved in high elevation adaptation, while blue dots represent potential SNPs involved in lowland adaptation. [file ECE3-15-e71846-s002.png]
